# The effect of bariatric surgery on the expression of gastrointestinal taste receptors: A systematic review

**DOI:** 10.1007/s11154-023-09865-7

**Published:** 2024-01-11

**Authors:** Rosalind Walmsley, Lynn Chong, Michael W. Hii, Robyn M. Brown, Priya Sumithran

**Affiliations:** 1 Department of Medicine, St Vincent’s Hospital Melbourne, University of Melbourne, Parkville, VIC 3052 Australia; 2 Department of Surgery, St Vincent’s Hospital Melbourne, University of Melbourne, Victoria, Australia; 3 Department of Pharmacology and Biochemistry, University of Melbourne, Victoria, Australia; 4 Department of Surgery, Central Clinical School, Monash University, Victoria, Australia; 5 Department of Endocrinology and Diabetes, Alfred Health, Victoria, Australia

**Keywords:** Taste preference, Bariatric surgery, Taste receptors, Nutrient sensing

## Abstract

Gastrointestinal nutrient sensing via taste receptors may contribute to weight loss, metabolic improvements, and a reduced preference for sweet and fatty foods following bariatric surgery. This review aimed to investigate the effect of bariatric surgery on the expression of oral and post-oral gastrointestinal taste receptors and associations between taste receptor alterations and clinical outcomes of bariatric surgery. A systematic review was conducted to capture data from both human and animal studies on changes in the expression of taste receptors in oral or post-oral gastrointestinal tissue following any type of bariatric surgery. Databases searched included Medline, Embase, Emcare, APA PsychInfo, Cochrane Library, and CINAHL. Two human and 21 animal studies were included. Bariatric surgery alters the quantity of many sweet, umami, and fatty acid taste receptors in the gastrointestinal tract. Changes to the expression of sweet and amino acid receptors occur most often in intestinal segments surgically repositioned more proximally, such as the alimentary limb after gastric bypass. Conversely, changes to fatty acid receptors were observed more frequently in the colon than in the small intestine. Significant heterogeneity in the methodology of included studies limited conclusions regarding the direction of change in taste receptor expression induced by bariatric surgeries. Few studies have investigated associations between taste receptor expression and clinical outcomes of bariatric surgery. As such, future studies should look to investigate the relationship between bariatric surgery-induced changes to gut taste receptor expression and function and the impact of surgery on taste preferences, food palatability, and eating behaviour.

**Registration code in PROSPERO:** CRD42022313992

## Introduction

Taste perception serves mainly to identify nutrients and avoid toxins. It is a multifaceted process, comprising sensation of the flavour of a substance as it comes into contact with its corresponding taste receptor, followed by perception of that sensation as enjoyable, unpleasant, or neutral. This process is entwined with other sensory modalities, such as smell, texture, and temperature detection which combine to provide an overall perception of the flavour of a substance. Traditionally, five basic tastes have been described in humans – sweet, sour, bitter, salty, and umami. More recently, research has demonstrated that fats stimulate a different class of receptors in a similar manner [1].

Each basic taste is detected by specific flavour receptors located on taste cell membranes in taste buds of the oral cavity [1]. Three types of mammalian taste cells have been identified. Type I cells are the most abundant, comprising more than half of all cells in each taste bud, and provide glial-like support to other taste cells [2]. Type II taste cells are the main chemosensory cells for the transduction of sweet, umami, fatty, and bitter flavours. Type III taste cells make up just 2–20% of cells in each taste bud and vary according to anatomical position in the oral cavity [1]. Although the specific receptors for sour and salt taste are still uncertain, nerve conduction studies in rodents show that sour flavours are sensed by type III taste cells [3], and salt taste is likely transmitted via either type I glial-like support cells [2] or a subtype of type II taste cells [4]. Each taste receptor subtype is structurally and functionally distinct. Sweet (T1R2, T1R3), bitter (T2Rs), and umami (T1R1, T1R3) taste receptors are G protein-coupled receptors (GPCRs). On lingual taste cells T1R2 and T1R3 heterodimerise to detect sweet flavours, and T1R1 and T1R3 heterodimerise to recognise umami flavours. Twenty-five T2R bitter taste receptors have been discovered in humans, each functioning as a monomer on lingual taste cells to recognise a wide variety of bitter tasting compounds and potential toxins. These GPCRs signal via a common intracellular signalling pathway involving α-gustducin, phospholipase C β2 (PLCβ2), and transient receptor potential cation channel subfamily M member 5 (TRPM5), leading to the release of adenosine triphosphate (ATP) following stimulation by ingested nutrients [1]. Some fatty acid taste receptors (G protein-coupled receptor 40 [GPR40]/ Free fatty acid receptor 1 [FFAR1] in mice and G protein-coupled receptor 120 [GPR120]/ Free fatty acid receptor 5 [FFAR4] in humans) are also GPCRs that trigger TRPM5 activation and intracellular calcium release to exert their effects [5]. Candidate salt [6] and sour [3, 7] receptors are ion channels, and other sweet (sodium-glucose transporter 1 [SGLT1]) and fatty acid (cluster of differentiation 36 [CD36]) receptors are nutrient transporters, where the presence of intracellular tastants triggers intracellular calcium accumulation [8] or transient elevation of ATP [1] and taste cell membrane depolarisation.

Stimulation of all taste receptors on lingual taste cells eventually results in activation of sensory neurons within branches of the facial, glossopharyngeal and vagus cranial nerves [9]. In humans, these nerves transmit flavour signals via the nucleus of the solitary tract to the gustatory cortices in the anterior insula and frontal operculum and mesocorticolimbic regions, such as the ventral tegmental area and nucleus accumbens, resulting in taste perception and the creation of taste preferences, which guide eating behaviours [9]. Lingual taste cells also secrete glucoregulatory and appetite-related hormones, such as glucagon-like peptide-1 (GLP-1), glucagon, ghrelin, cholecystokinin (CCK) and peptide YY (PYY), following stimulation of taste receptors [10]. Although not completely understood, it is thought that these peptides function as autocrine and paracrine signals to modulate the sensitivity of taste perception at the taste bud level [11].

The same taste receptors responsible for lingual taste transduction of bitter (T2Rs), sweet (T1R2 and T1R3), umami (T1R1 and T1R3), and fatty acid (CD36, GPR40/FFAR1 and GPR120/FFAR4) tastants are also found in the gastric, small bowel, and colonic mucosa [12]. While monomers of the T1R class of taste receptors heterodimerise in the oral cavity to detect sweet and umami taste, T1R1, T1R2, and T1R3 exist and function individually in the gastrointestinal tract [12]. Stimulation of these receptors, as well as bitter and fatty acid monomers leads to release of incretins (GLP-1, Glucose-dependent insulinotropic polypeptide [GIP]) and other satiety hormones (PYY, CCK) [13–16]. Stimulation of sweet receptors also results in the upregulation of glucose transporters SGLT1 and glucose transporter 2 (GLUT2) on neighbouring enterocytes [17–19]. Intragastric infusion of fats and sugars can induce preference for different orally coupled flavoured solutions in rodents [20–22]. Likewise, intragastric infusion of bitter compounds can induce flavour aversions to orally-coupled substances, even if the oral substance was initially preferred by the animal [23]. Furthermore, unlike wild-type mice, SGLT1 gene knockout mice develop no preference for oral flavours paired with intragastric glucose infusions compared to those paired with water infusions in two-bottle choice tests [24]. *In vivo* calcium imaging of vagal neurons demonstrates attenuated activity in response to intra-intestinal glucose or fat infusions in SGLT1 knockout and GPR40/120 double knockout mice, respectively [20]. These studies suggest that gut taste receptors play an important role in the development of food preferences.

Bariatric surgery results in sustained weight loss and metabolic improvements [25–27]. Alterations of food preferences and taste perception are frequently reported [28–31]. This is supported by functional MRI studies, which have demonstrated blunting of response in the mesolimbic reward pathway following ingestion of calorie-dense foods in patients who have undergone gastric bypass and sleeve gastrectomy [30–32]. Many of the changes occurring after bariatric surgery, including increases in post-prandial release of PYY and GLP-1, changes to vagal signalling [33] and food preferences parallel known or proposed functions of gastrointestinal taste receptors. Furthermore, expression of gut taste receptors is altered in states of metabolic dysfunction such as type 2 diabetes [34, 35] and obesity [36–40] that are ameliorated by bariatric surgery. As such, a potential role for these receptors in metabolic and food preference changes observed after bariatric surgery is plausible.

This review aims to evaluate and synthesise the current literature on the effect of bariatric surgery on the expression of oral and post-oral gastrointestinal taste receptors. Its secondary aim is to explore the association between these taste receptors and clinical outcomes of bariatric surgery.

## Methods

### Study design

This review was designed in accordance with the Preferred Reporting Items for Systematic Reviews and Meta-analyses (PRISMA) 2020 explanation and elaboration guidelines [41]. A search of electronic databases was conducted in March 2022. Duplicate publications were excluded using Endnote electronic reference manager [42]. Screening was conducted by two independent reviewers using Covidence [43]. Disputes regarding inclusion of studies were resolved by consensus. The reference lists of relevant review articles were searched for potential additional studies. Data was extracted by a single reviewer. Due to the heterogeneity of the included studies conduction of a meta-analysis was not possible.

### Search strategy

A search of Medline (Ovid, 1946–2022), Embase (Ovid, 1974–2022), Emcare (Ovid 1995–2022), APA PsychInfo (Ovid, 1806–2022), Cochrane Library, and CINAHL (EBSCOhost) electronic databases was performed on 10th of March 2022 using a combination of keywords and MeSH terms (Table 1). No date or language limits were applied. Online software ‘Polyglot’ was used to translate search syntax across databases [44].

**Table 1:** Search string for Ovid (Medline) (searched March 10, 2022)

“bariatric surgery [MeSH]”; OR “bariatric surgery” OR “metabolic surgery” OR “obesity surgery” OR “gastric bypass*” OR “RYGB” OR “sleeve gastrectom*” OR “SG” OR “single anastomos*” OR “one anastomos*” OR “OAGB” OR “SAGB” OR “gastric band*” OR “LAGB” OR “vertical band* gastroplast*” OR “duodenal switch*” OR “biliopancreatic diversion*” OR “BPD” OR “duodenal jejunal bypass*” OR “DJB”; AND “G-Protein-Coupled [MeSH]” OR “Taste [MeSH]” OR “Taste Buds [MeSH]”; OR “taste” OR “taste recept*” OR “chemosens*” OR “taste percept*” OR “taste detect*” OR “nutrient sens*” OR “TASR”	

The publication of research identifying the additional function of the glucose sensor SGLT1 as a sweet taste receptor occurred during the data extraction phase. A supplementary search of the aforementioned databases was conducted on the 25th of May 2022 with the following search terms added to the strategy detailed in Table 1: “SGLT*” or “glucose absor*” or “GLUT*” or “glucose transport*”.

### Eligibility criteria

Human or animal English-language studies were eligible for inclusion. No date restriction was applied.

Inclusion criteria:

- Full text available in English
- Any type of bariatric surgery
- Reports data on tissue analysis of oral or post-oral gastrointestinal taste receptors
- Reports original data, including randomised controlled trials, cohort studies, case reports and case series.

Exclusion criteria:

- Analysis of receptors not previously demonstrated to be involved in taste signalling pathways, e.g. bile acid receptors, non-SGLT1 glucose transporters.
- Artificial tissues – e.g. organoids
- Uncontrolled studies (those without a non-operated or sham-operated group for comparison).
- Systematic reviews and meta-analyses, conference abstracts, editorials, and letters-to-the-editor.

### Data collection process

The following information was extracted from the included studies: publication year, study type, population, sample size, type of bariatric surgery performed, method of control, participant age, sex, baseline weight and diabetes status, type and subclass of taste receptor analysed, anatomical location of taste receptor, method of tissue analysis, change (pre-/post-surgery) or difference (surgery vs non-surgery control) in expression or function of taste receptor, time between bariatric surgery and analysis, taste perception or food preference test methods and results, change (pre-/post-surgery) or difference (surgery vs non-surgery control) in weight, fat mass, blood glucose and lipids and circulating gastrointestinal hormone levels.

### Outcomes

The primary outcome is magnitude of change in the tissue expression of oral or post-oral gastrointestinal taste receptors following bariatric surgery.

The secondary outcomes are associations between oral or post-oral gastrointestinal taste receptors and the following clinical outcomes of bariatric surgery; food preference, taste perception, weight loss, fat loss, circulating lipid, glucose, or gastrointestinal hormone levels.

### Risk of bias assessment

The methodological quality and risk of bias of included human studies were assessed using the National Institute of Health (NIH) quality assessment tool for observational cohort and cross-sectional studies [45], and the NIH tool for before-after (pre-post) studies with no control groups [46]. Items #8 (concerning range of exposure) and #10 (repeated exposure assessment) were excluded from the cohort and cross-sectional study assessment due to irrelevance to the review question, as was item #12 (pertaining to statistical analysis of interventions conducted on the group level) on the pre-post study assessment tool.

Animal studies were assessed for internal validity using the SYRCLE risk-of-bias tool, which uses 10 criteria to assess six types of bias: selection, performance, detection, attrition, reporting, and ‘other sources of bias’ [47]. For the purpose of this review ‘other sources’ refers to possible biases resulting from funding sources and conflicts of interest. When assessing selection bias (criterion #3) study groups were considered to be similar at baseline if the species, genotype, age, sex, body weight, and food intake did not significantly differ between groups.

Quality assessments were carried out by two authors independently and discrepancies resolved by consensus.

## Results

### Study selection and characteristics

**Fig. 1:**
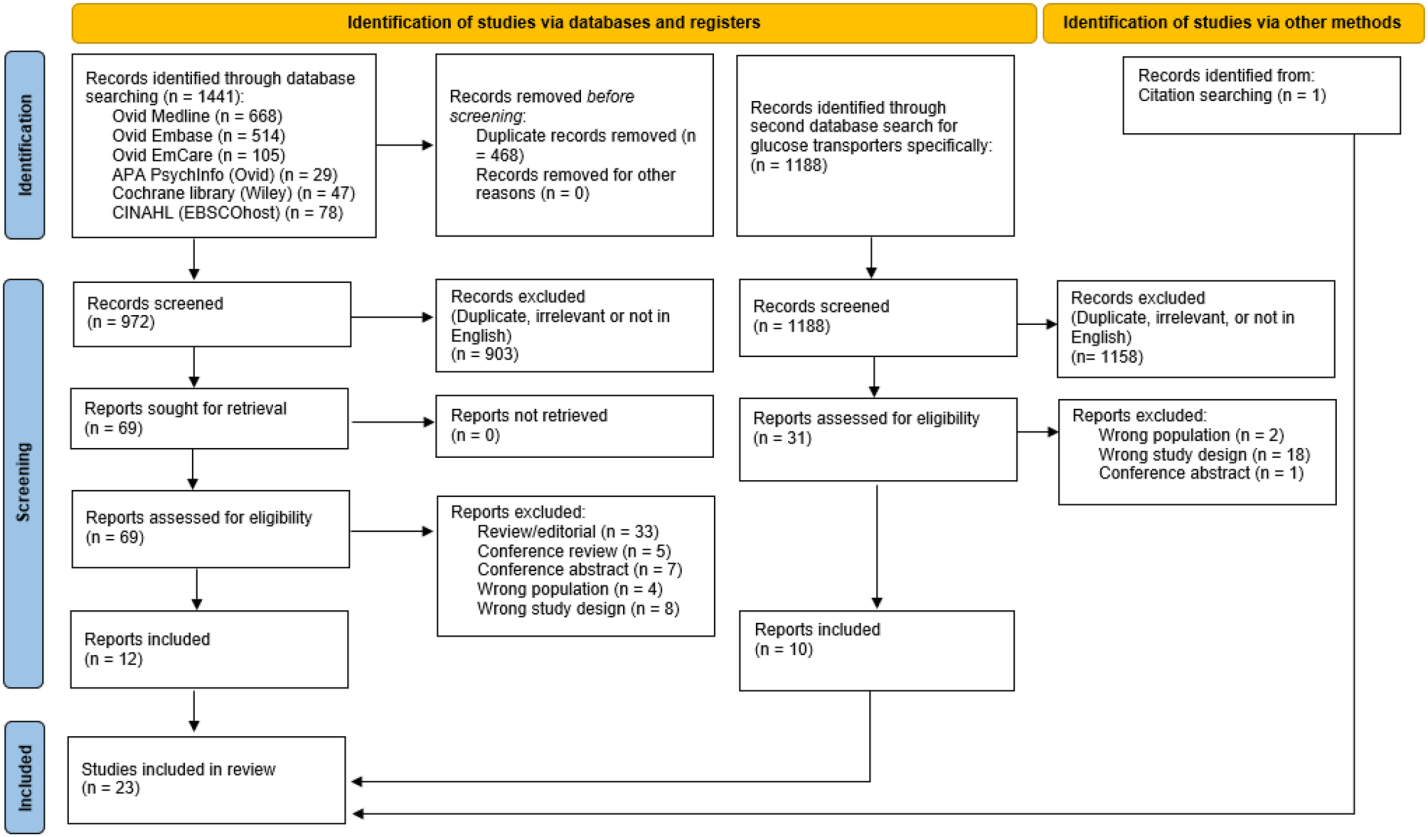
PRISMA flow diagram illustrating the process of the literature search

The search identified 23 studies for inclusion (Fig. 1), comprising two human studies and 21 animal studies. The two human studies included one longitudinal cohort [48] and one cross-sectional study [49]. The key characteristics are outlined in Table 2. In total, 29 participants underwent Roux-en-Y gastric bypass (RYGB) [48, 49] and 10 participants underwent laparoscopic adjustable gastric banding (LAGB) [48]. Participants had a mean age range of 42 to 52 years, 67% were female and none had diabetes. Time since surgery ranged from four months to 12 years. Taste receptor expression was analysed by quantitative polymerase chain reaction (qPCR) in oral (fungiform papillae) in one study [48], and jejunal/proximal alimentary limb mucosa in the other [49].

**Table 2:** Characteristics of included human studies

**Study**	**Design**	**Surgery**	**Control group**	**Post-operative timing of tissue collection**	**Mean age** **(years)**	**Female: male ratio**	**Mean Weight**	**History of diabetes**	**Taste receptor** **analysed**	**Location of tissue analysed**	**Method of analysis**	**Degree & direction of change after bariatric surgery**
Pepino et al. (2014) [48]	Pre-post cohort	RYGB (n = 17) LAGB (n = 10)	Pre-surgery (within-person)	4–6 months (once 20% total weight loss reached)	RYGB 42 ± 8 LAGB 47 ± 14	Female 100%	Baseline RYGB: 123.8 ± 19.7 kg BMI 46.3 ± 7.7 kg/m^2^ Baseline LAGB: 127.1 ± 31.0 kg BMI 48.5 ± 10.5 kg/m^2^	No	α-gustducin PLCB2 T1R3 T1R2 T1R1	Tongue (fungiform papillae)	qPCR (mRNA)	↓ α-gustducin (× 3) post-RYGB and post-LAGB <-> PLCβ2, T1R3, T1R2, T1R1
Nguyen et al. (2014) [49]	Cross-sectional	RYGB (n = 12)	Obese (n = 14) Lean (n = 11)	Mean 4.5 (range 2–12) years	RYGB 52 ± 2 Obese: 52 ± 2 Lean: 44 ± 6	RYGB: 7F: 4M Obese: 8F: 5M Lean: 1F: 10M	BMI at time of analysis RYGB: 31 ± 2 kg/m^2^ Obese: 42 ± 4 kg/m^2^ Lean: 25 ± 1 kg/m^2^	No	T1R2 SGLT1	Proximal alimentary limb	qPCR (mRNA)	Fasting: ↑ SGLT1 <-> T1R2 Post luminal sugar infusion: ↓ T1R2 <-> SGLT1 post-RYGB but ↓ in obese/lean

↓ decreased gene or protein expression, ↑ increased gene or protein expression, <-> no significant change, *F* female, *M* male, *BMI* body mass index, *RYGB* Roux-en-Y gastric bypass, *LAGB* laparoscopic adjustable gastric banding, *qPCR* quantitative polymerase chain reaction, *mRNA* messenger RNA

Animal studies were conducted in rats (13 studies), mice (seven studies) or both (one study). The bariatric procedure was RYGB in nine studies [50–58], duodenal-jejunal bypass (DJB) in four studies [59–62], sleeve gastrectomy (SG) in seven studies [50, 63–67], and entero-gastric anastomosis (EGA) procedures [35], single-anastomosis duodenal-jejunal bypass (SA-DJB) [68], and ileal interposition (IIP) [69] in one study each. Five studies included analysis of animals with diabetes or insulin resistance [54, 61, 62, 67, 69]. Time between surgery and tissue harvest ranged between 11 days and six months. Taste receptor quantification was carried out using PCR for mRNA analysis in six studies [35, 50, 54, 57, 58, 61], protein analysis techniques (such as Western blotting, immunohistochemistry, or mass spectrometry) in another five [52, 53, 56, 68, 69], or both in 10 studies [51, 55, 59, 60, 62–67]. Key characteristics of animal studies are outlined in Table 3.

**Table 3:** Characteristics of included animal studies

**Study**	**Population**	**Baseline characteristics**	**Surgery**	**Control group**	**Post-operative timing of tissue collection**	**Taste receptor** **analysed**	**Location of tissue analysed**	**Method of tissue analysis**	**Degree & direction of change after bariatric surgery**
Steensels et al. (2017) [58]	Mice C57BL/6 WT & α-gustducin-KO (n = 7–9 per genotype, per intervention)	Male Western diet pre-op	RYGB	Sham surgery (ad libitum & pair fed)	7 weeks	T1R3 SGLT1 LPAR5 FFAR2 FFAR3 FFAR4 α-gustducin	AL BPL Colon	qPCR (mRNA)	↑ LPAR5 in BP limb but not AL in WT mice ↑ LPAR5 in AL only and ↓ T1R3 in BP limb in α-gustducin-KO mice <--> SGLT1 or FFAR4 in BPL or AL of both genotypes ↓ FFAR2 in colon of α-gustducin-KO mice ↓ FFAR3 in colon of both genotypes
Le Gléau et al. (2021) [35]	Mice C57BL/6	Male DIO	EGA (n = 16)	Sham surgery (n = 14)	4 weeks	α-gustducin	Distal jejunum (AL) Ileum (CC)	qPCR (mRNA)	↑ α-gustducin in distal jejunum (A limb) <--> in ileum
Jiang et al. (2022) [62]	Wistar Rats N = 40	Male T2DM	DJB (n = 10)	Sham surgery (n = 10) & Non-operated lean, euglycaemic (n = 10)	6 weeks	T1R2 T1R3 SGLT1	AL	qPCR (mRNA) Western Blot (protein)	↓ T1R2, T1R3 and SGLT1 mRNA & protein The ↓ in SGLT1 was relative to T2DM-sham bringing levels back in line with those of euglycaemic control
Peiris et al. (2018) [57]	Mice C57BL/6	Male DIO (mean weight 41.5g)	RYGB	Sham surgery HFD (n = 5–7) & Post-DIO VLCD weight loss (n = 5–7) & Non-operated chow-fed lean (n = 5–7)	12 weeks	FFAR1/GPR40 FFAR2/GPR43 FFAR3/GPR41 FFAR4/GPR120 GPR84 GPR119 LPAR5/GPR92/93 T1R3 SGLT1	Colon	qPCR (mRNA)	↑ FFAR1, FFAR3, GPR84, & GPR119 mRNA post-RYGB <--> FFAR2, T1R3, SGLT1, FFAR4 and LPAR5 mRNA
Yu et al. (2020) [68]	Goto-Kakisaki Rats (n = 12)	Male	SA- DJB (n = 6)	Sham surgery (n = 6)	8 weeks	FFAR2/GPR43 FFAR3/GPR41	‘intestinal’ *Not further specified*	Western Blot (protein) IHC (protein) Immunofluorescence (protein)	↑ FFAR2 and FFAR3 protein post-SA-DJB
Hankir et al. (2017) [56]	Wistar Rats (n = 160)	Male DIO-HFD	RYGB	Sham surgery (low fat diet & body weight matched)	*Not specified*	OEA PPAR- α	BPL AL CC	Mass spectrometry with liquid chromatography (protein)	↑ post-prandial OEA in proximal AL + CC ↓ post-prandial OEA in BP limb OEA production PPAR-a mediated (suppressed when pharmacologically inhibited)
Fruhbeck et al. (2022) [67]	Wistar Rats Zucker fa/fa	Male DIO vs genetic Obesity with insulin resistance	SG (n = 7)	Sham surgery (n = 7) & VLCD (n = 9)	4 weeks & 4 months	CD36	Tongue	qPCR (mRNA) Western Blot (protein) IHC (protein)	↓ CD36 mRNA & protein at 4 weeks but not 4 months
Bueter et al. (2011) [55]	Wistar Rats	Male 425-450g	RYGB	Sham surgery	60 days	T1R3 T1R2	BPL AL CC	qPCR (mRNA) Western Blot (protein)	↓ T1R2 mRNA in BP limb only (<--> T1R2 mRNA in A limb or CC) ↓ T1R2 protein levels in both A + BP limb <--> T1R2 protein in CC <--> T1R3 mRNA in all 3 regions ↓ T1R3 protein in A limb only
Hutch et al. (2020) [66]	Long-Evans Rats C57BL/6 WT mice PPAR-α KO mice GPR119 KO mice CD36 KO mice	Male DIO	SG	Sham surgery	10–12 weeks	OEA CD36 GPR119 PPAR- α	Duodenum Distal Jejunum Ileum	qPCR (mRNA) Mass spectrometry (protein)	↑ CD36, GPR119 mRNA in duodenum of WT post-SG but <-> in jejunum or ileum <--> PPAR- α mRNA all 3 regions ↑ OEA protein in duodenum of WT mice and rats post-SG but <-> in jejunum or ileum
Yan et al. (2013) [60]	Mice C57BL/6 (n = 36)	Male	DJB (n = 18)	Sham surgery (n = 18)	2 weeks 1 month 2 months	SGLT1	Gastrojejunal segment (A limb) Ileum (CC)	qPCR (mRNA) Western Blot (protein)	↓SGLT1 mRNA and protein in A limb by 46% at 1 month and by 42% at 2 months ↓SGLT1 mRNA but not protein in ileum (CC) by 40% at 1 month and by 30% at 2 months <--> SGLT1 mRNA or protein in either region at 2 weeks
Jurowich et al. (2013) [61]	Lewis Rats	Male T2DM	DJB	Sham surgery	3 weeks	SGLT1	Duodenum (BPL) Jejunum (AL) Ileum (CC)	qPCR (mRNA)	↓SGLT1 mRNA by 50% in A limb <--> SGLT1 mRNA in BPL or CC
Kaufman et al. (2019) [54]	Sprague–Dawley Rats	Male Insulin-resistant	RYGB	Sham surgery	21 days	CD36	Distal Jejunum	qPCR (mRNA)	↓ CD36 mRNA post-RYGB
Kim et al. (2015) [59]	Sprague–Dawley Rats (n = 25)	Male 380-400g	DJB (n = 15)	Sham surgery (n = 10)	5 weeks	SGLT1	AL	qPCR (mRNA) Western Blot (protein) IHC (protein)	↑ SGLT1 mRNA + protein
Taqi et al. (2010) [53]	Sprague–Dawley Rats (n = 16)	Male 350-400g	RYGB (n = 8)	Sham surgery (n = 8)	14 days	SGLT1	AL BPL CC	Western Blot (protein)	↓ SGLT1 protein in AL <--> SGLT1 protein in BPL or CC
Ren et al. (2022) [65]	Mice C57BL/6 (n = 60)	Male	SG (n = 30)	Sham surgery (n = 18) & Non-operated control (n = 12)	14 days 30 days	SGLT1	Distal Jejunum Proximal ileum	qPCR (mRNA) Immunofluorescence (protein)	↓ SGLT1 mRNA and protein in both jejunum and ileum at 14 and 30 days
Stearns et al. (2009) [51]	Sprague–Dawley Rats	Male 300-325g	RYGB (n = 23)	Sham surgery (n = 22)	3 weeks	SGLT1	AL	qPCR (mRNA) Western Blot (protein)	↑SGLT1 mRNA in Western blot demonstrated post-transcriptional modifications to SGLT1 with the addition of different deglycosylated species
Pérez-Arana et al. (2022) [52]	Wistar Rats (n = 36)	Male 200-220g	RYGB (n = 12)	Sham surgery (n = 12) & Non-operated fasting (n = 12)	12 weeks 24 weeks	SGLT1	AL	Immunostaining (protein)	↑ SGLT1 protein in AL at both timepoints
Xia et al. (2019) [63]	Mice C57BL/6	Male Non-obese	SG (n = 6)	Sham surgery (n = 6)	2 weeks 1 month 2 months	SGLT1	Residual stomach Duodenum Proximal jejunum Distal jejunum Proximal ileum	qPCR (mRNA) Immunofluorescence (protein)	↓ SGLT1 mRNA and protein at 2W + 1M then ↑ SGLT1 mRNA and protein at 2M in all regions
Du et al. (2018) [64]	Mice C57BL/6 (n = 137)	Male DIO	SG (n = 48)	Sham surgery (n = 28) & Non-operated HFD (n = 29) & Non-operated pair-fed (n = 10)	8 weeks	SGLT1	Jejunum	qPCR (mRNA) Western Blot (protein) IHC (protein)	<--> SGLT1 mRNA in jejunum post-SG ↑ SGLT1 protein in jejunum post- SG compared to non-operated HFD and sham surgery controls
Jurowich et al. (2015) [69]	Lewis Rats (n = 27)	Male 180-200g Insulin resistant	IIP (n = 9)	Sham surgery (n = 9) & Non-operated HFD (n = 9)	6 weeks	SGLT1	Interposed ileal segment	IHC (protein)	↑ SGLT1 protein 1.9-fold in interposed ileal segment
Cavin et al. (2016) [50]	Wistar Rats	Male DIO (675 ± 50 g)	RYGB & SG	Sham surgery	14 days 40 days	SGLT1	BPL AL & Jejunum post- SG	qPCR (mRNA)	↑ SGLT1 mRNA in AL at day 40 but not day 14 <--> SGLT1 in BP limb at either timepoint <--> SGLT1 in jejunum post-SG at either timepoint

*WT* wild-type, *DIO* diet-induced obesity, *T2DM* type 2 diabetes mellitus, *HFD* high-fat diet, *VLCD* very low calorie diet, *RYGB* Roux-en-Y gastric bypass, *EGA* entero-gastric anastomosis, *DJB* duodenal-jejunal bypass, *SG* sleeve gastrectomy, *SA-DJB* single-anastomosis duodenal-jejunal bypass, *IIP* ileal interposition, *BPL* biliopancreatic limb, *AL* alimentary limb, *CC* common channel, *qPCR* quantitative polymerase chain reaction, *mRNA* messenger RNA, *IHC* immunohistochemistry, *KO* knockout, ↓ decreased gene or protein expression, ↑ increased gene or protein expression, <-> no significant difference

### Risk of bias

**Fig. 2:**
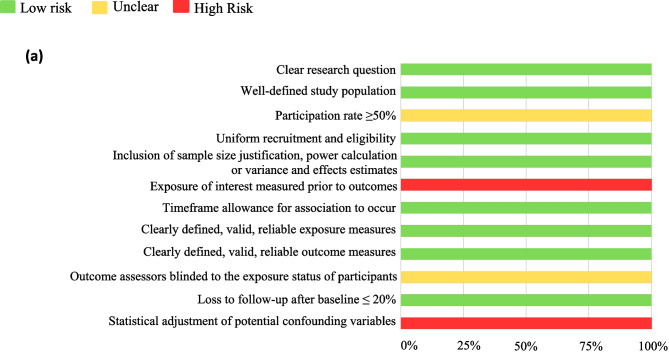

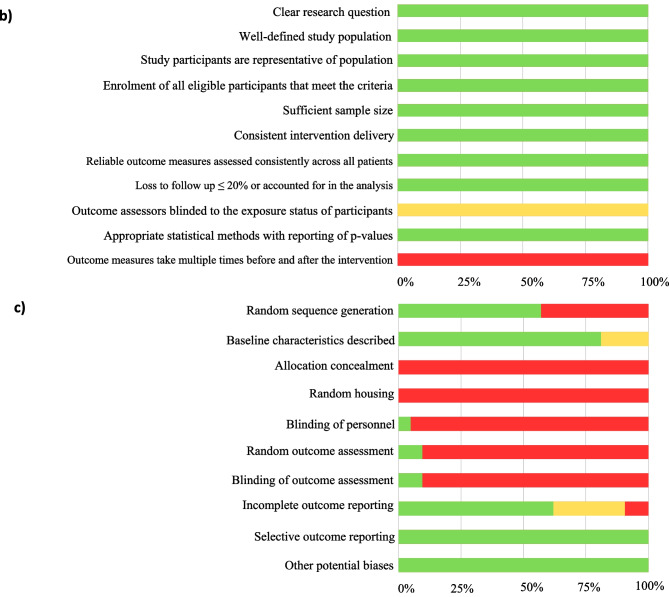
Summary of quality assessment results for: cross-sectional and cohort studies (**a**), pre-post observational studies without a control group (**b**), and animal models (**c**)

Quality and risk-of-bias assessments are presented in Fig. 2. The overall quality of the human studies was rated as good. The internal validity of the 21 animal studies was limited in each case by lack of reporting on key bias reduction measures, therefore, most items in the risk-of-bias tool were assessed as ‘high risk’ due to omission. Although 12 studies mentioned randomization of group allocation, no study specified the method of randomisation. Similarly, three studies reported using a form of ‘blinding’ to reduce bias but provided minimal detail on the process. Most studies provided sufficient detail regarding pre-intervention characteristics of animals, and all studies adequately addressed the risk of reporting bias (Fig. 2c). The risk of conflicting interest was low for all articles.

### Changes to the gene or protein expression of taste receptors induced by bariatric surgery

#### Sweet taste receptors

Lingual taste perception of sugars, artificial sweeteners and other sweet compounds occurs primarily through the activation of the T1R2/T1R3 heterodimer [70], although an alternative pathway utilising SGLT1 that is specific for glucose detection also exists [71]. All three receptors have been co-localised to enteroendocrine cells of the gastrointestinal tract [21, 72], where SGLT1 transduces information regarding the presence of glucose and its analogues [21, 24, 73] and T1R3 (with or without T1R2) responds only to artificial sweeteners [21].

Two human [48, 49] and 15 animal studies [50–53, 55, 57–65, 69] examined the effect of bariatric surgery on the sweet taste receptors SGLT1 (14 studies), T1R2 (four studies), and T1R3 (five studies).

##### SGLT1

One human study reported an increase in basal alimentary limb SGLT1 mRNA expression after RYGB, which was unchanged following a 30-min luminal glucose infusion [49].

Of the animal studies, five reported an increase in SGLT1 mRNA and protein expression in the alimentary limb after RYGB [50–52], and DJB [59] and in the interposed ileal segment after IIP [69]. No change to mRNA levels were observed in the alimentary limb of one study [58] or the biliopancreatic limb [50, 58], or colon [57] after RYGB. Two studies reported no change to SGLT1 mRNA in jejunal mucosa after SG [50, 64]. However, one of these studies did find a reduction in jejunal SGLT1 protein expression [64]. Five studies observed a decrease in the mRNA or protein of SGLT1 after bariatric surgery (n = 1 alimentary limb following RYGB, n = 3 alimentary limb following DJB, n = 1 jejunum and ileum following SG) [53, 60–62, 65]. Additionally, one study reported a decrease in the abundance of stomach, duodenal, jejunal and ileal SGLT1 mRNA and protein at 2 and 4 weeks after SG, followed by an increased expression above baseline at 8 weeks [63].

##### T1R2

Two human studies found no change in the mRNA expression of oral [48] or basal proximal alimentary limb [49] T1R2 after RYGB. However, following luminal glucose infusion, T1R2 mRNA expression decreased in the alimentary limb and remained unchanged in non-operated control groups [49]. There was no change to oral T1R2 mRNA expression following LAGB [48].

Two animal studies reported a decrease in the mRNA and protein expression of T1R2 in the alimentary limb [55, 62] and biliopancreatic limbs [55] after RYGB and DJB. No difference in the mRNA or protein levels of T1R2 in the common channel after RYGB was observed [55].

##### T1R3

No studies examined intestinal T1R3 mRNA or protein expression in humans after bariatric surgery, but one reported no change to oral T1R3 mRNA expression after RYGB and LAGB [48].

Small intestinal T1R3 mRNA and protein expression was decreased following RYGB [55, 58] and DJB [62] in animals. In one of these studies, the reduction was observed only in α-gustducin gene knockout (α-gustducin-KO) mice and not their wild-type counterparts [58]. In the other RYGB study, no change in mRNA expression of T1R3 was found, but T1R3 protein levels were reduced in the alimentary limb when compared to sham-operated control rats [55]. One study reported no change to colonic mRNA expression after RYGB [57].

#### Amino acid taste receptors

Lingual umami taste transduction occurs primarily through amino acid activation of the T1R1/T1R3 heterodimer [70]. Both T1R1 and T1R3 have been found along the entire length of the gastrointestinal tract individually, however co-localization has previously only been shown in the duodenal mucosa [72]. The T1R3 monomer is shared between the umami and sweet taste pathways and as such, has been discussed in the earlier section on *sweet taste receptors*.

Lingual T1R1 mRNA expression was not changed in humans after RYGB or LAGB [48]. No study analysed either protein or mRNA expression of T1R1 in the gut after bariatric surgery.

Many other receptors have been implicated in gastrointestinal cell-surface amino acid sensing following ingestion of dietary proteins [74, 75]. Of these, only lysophosphatidic acid receptor 5 (LPAR5, also known as G protein-coupled receptor 92/93 [GPR92/93]) has been investigated for changes following bariatric surgery (two studies) [57, 58]. After RYGB, expression of LPAR5 mRNA increased in the biliopancreatic limb of obese wild-type mice and in the alimentary limb of their α-gustducin-KO counterparts [58]. Colonic LPAR5 mRNA levels were unchanged in mice after RYGB compared to sham-operated, lean, and obese controls [57].

#### Fatty acid receptors

Taste perception of dietary fats has been proposed as a sixth basic taste [1, 76] but the mechanism by which fatty acid taste perception occurs is not fully elucidated [1]. Several fatty acid receptors and intracellular signalling mediators located on or in oral taste cells or enteroendocrine cells have been proposed as candidate fat taste receptors. These include fatty acid translocase (CD36), free fatty acid receptors 1 – 4 (FFAR1/GPR40, free fatty acid receptor 2 [FFAR2]/ G protein-coupled receptor 43 [GPR43], free fatty acid receptor 3 [FFAR3]/ G protein-coupled receptor 41 [GPR41], FFAR4/GPR120), G protein-coupled receptor 119 [GPR119], G protein-coupled receptor 84 [GPR84], the nuclear receptor peroxisome proliferator-activated receptor-alpha (PPAR- α) and its ligand oleoylethanolamide (OEA) [77–79].

Seven animal studies investigated the effect of bariatric surgery on the mRNA or protein expression of gastrointestinal fatty acid receptors [54, 56–58, 66–68]. FFAR3 mRNA and protein levels were increased in the intestine (location not specified) after SA-DJB [68] and in the colon after RYGB [57]. FFAR2 protein levels were increased following SA-DJB [68] but FFAR2 mRNA levels were unchanged after RYGB [57]. Colonic FFAR1/GPR40, GPR84 and GPR119 mRNA expression was increased post-RYGB [57]. Conversely, one study found a reduction in FFAR3 mRNA expression in the colon of wild-type and α-gustducin-KO mice subjected to RYGB, and a reduction in FFAR2 expression in α-gustducin-KO mice alone [58]. No change to FFAR4 mRNA expression in the alimentary or biliopancreatic limb [58] or colon [57] of RYGB mice was observed.

Post-prandial levels of OEA increased in the alimentary limb and common channel and decreased in the biliopancreatic limb of mice following RYGB [56]. Mice that underwent SG had increased duodenal mRNA expression of CD36, GPR119 and increased intracellular production of OEA but no change to these molecules was found in the jejunum or ileum [66]. PPAR-α mRNA expression remained unchanged in the duodenum, jejunum, and ileum post-SG [66]. SG decreased lingual CD36 mRNA and protein [67] and RYGB decreased jejunal CD36 mRNA [54] in rats.

#### Alpha-gustducin & other G-protein-coupled receptor (GCPR) intracellular taste signalling machinery

Alpha-gustducin is a primarily taste-specific G-protein alpha subunit responsible for the coupling of sweet, bitter, and umami GPCRs with intracellular second messenger enzyme systems, leading to the opening of cation channels and calcium influx necessary for the release of neurotransmitters (e.g. the purinergic agonist ATP) and peptides (e.g. GLP-1, GIP, PYY) from oral taste cells and enteroendocrine cells [70, 74]. PLCβ2 is an intracellular taste signalling molecule involved in the same calcium influx pathway [70].

One human [48] and one mouse study [35] assessed changes to mRNA expression of intracellular taste signalling molecules. Both studies looked for changes to α-gustducin [35, 48], and one for changes to PLCβ2 [48]. One other study assessed the function of α-gustducin after RYGB using gene knockout mice, which is discussed in earlier sections [58].

Pepino et al. observed a threefold decrease of α-gustducin mRNA and no change to PLCβ2 mRNA in oral fungiform papillae in humans after RYGB and LAGB [48]. In mice, an increase in the abundance of α-gustducin mRNA in the distal jejunum and ileum was reported following EGA [35].

#### Bitter, salty & sour taste receptors

No study has reported changes to the gene or protein expression of bitter, salty or sour taste receptors in the oral cavity or gastrointestinal tract of humans or animals.

### Association between changes to the gene or protein expression of taste receptors and taste perception and food preference after bariatric surgery

Of the 23 included studies, five assessed changes to taste perception and/or food preference after bariatric surgery alongside changes to the mRNA or protein expression of taste receptors. One was conducted in humans after RYGB and LAGB [48], with the other four assessing food preference and taste detection thresholds in rodents after RYGB [55, 56] and SG [66, 67].

All four studies that assessed differences in fat consumption compared to non-surgical comparators observed a reduced preference for and/or intake of fat after RYGB [48, 56], SG [66, 67], and LAGB [48]. The two studies that examined relationships between changes in fat preference and taste receptor expression found that in mice after SG [66] and RYGB [56], reduced preference for fat is associated with increased intestinal production of intracellular OEA [56] and mediated through PPAR-α activation [56, 66]. Knockout of CD36 attenuated the reduced preference for fat observed in mice after SG whereas GPR119 knockout did not influence eating behaviour [66].

Both studies analysing the impact of bariatric surgery on sweet food intake in humans [48] and rats [55] observed a decreased preference for, and intake of, sugar compared to control groups but neither examined relationships between changes in sweet preference and taste receptor expression. No effect of surgery was found on sweet taste detection thresholds [48].

Two studies assessed changes to intake or detection of umami in humans [48] or rodents [66], two studies assessed salty flavour [48, 55], and one study assessed sour flavour in rats [55], but no changes after bariatric surgery were found, and therefore associations with taste receptor changes were not tested. Details of the studies are outlined in Table 4.

**Table 4:** Food preference and taste perception changes after bariatric surgery

**Author (year)**	**Surgery**	**taste of interest**	**Test**	**Outcome**	**Taste receptor analysed**	**Analysis of association with taste receptor changes**
Pepino et al. (2014) [48]	RYGB LAGB	Sweet Fat Umami Salty	Food craving inventory Sweet taste questionnaire Fat preference questionnaire Taste detection thresholds (sucrose, glucose, salt, MSG) Above-threshold sensory function Forced-choice, paired-comparison preference test (sucrose & MSG) Sweet taste palatability test	↓ craving for sweets and fast food after RYGB and LAGB <--> craving for carbohydrates or high fat ↓ mood-altering effects of sweets post-RYGB than post-LAGB ↑ control of eating sweets post-RYGB and -LAGB ↓ frequency of fat intake post-RYGB and LAGB <--> taste perception of fat <--> for all 4 tastants post-RYGB and -LAGB ↓ perception of the sweetness of sucrose by 7% after surgery <--> perception of glucose, sodium chloride or MSG ↓ sucrose concentrations preferred after surgery <--> MSG concentration preference A negative association between changes in perceived sweetness intensity and the most preferred sucrose concentration ↓ palatability of sucrose from pleasant to unpleasant post-RYGB, but not post-LAGB,	α-gust PLCB2 T1R3 T1R2 T1R1	No
Hankir et al. (2017) [56]	RYGB	Fat	Two-choice diet (high-fat vs low-fat chow Two-bottle preference test (fat vs water)	↓preference for fat post-RYGB ↓ intake and preference for high fat emulsion post-RYGB ↑ intake and preference for very-low-fat (0.1% Intralipid) solutions post-RYGB	OEA PPAR- α	Blocking OEA, PPAR- α, vagal, or dorsal striatal dopamine-receptor signalling negated the effects of RYGB on fat intake and preferences
Fruhbeck et al. (2022) [67]	SG	Fat	Two-choice diet (ad libitum access to normal chow diet & high-fat diet)	↓ fat intake post-SG, especially 3–4 months post-surgery	CD36	No
Bueter et al. (2011) [55]	RYGB	Sweet Salty Bitter Sour	Two-bottle preference + 24 h intake (increasing concentrations of sucrose vs water) Two-bottle preference salty, bitter, sour tastes (vs water)	↓ sucrose intake at concentrations ≥ 10mM post-RYGB <--> sucrose intake at concentrations < 10mM ↓ calories consumed at every tested sucrose concentration post-RYGB <--> intake of sodium chloride, quinine hydrochloride or citric acid at any concentration post-RYGB	T1R2 T1R3	No
Hutch et al. (2020) [66]	SG	Fat	Macronutrient preference test (ad libitum food choice)	↓ fat and ↑ carbohydrate intake post-SG in wild-type mice SG-induced no change in macronutrient preference in PPARαKO mice. PPARαKO mice had overall higher preference for carbohydrates and less preference for fat compared with WT mice Protein intake was comparable between surgical groups & genotypes SG does not induce a food preference shift in CD36KO mice	OEA CD36 GPR119 PPAR- α	Loss of reduced fat/increased carbohydrate preference in CD36KO and PPARαKO mice GPR119KO maintained reduced preference for fat after SG

*RYGB* Roux-en-Y gastric bypass, *LAGB* laparoscopic adjustable gastric banding, *SG* sleeve gastrectomy, *MSG* monosodium glutamate, ↓ decreased preference, ↑ increased preference, <-> no change or non-significant change to preference, *KO* knock out, *WT* wild-type

### Association between gene or protein expression of taste receptors and clinical outcomes after bariatric surgery

Studies that investigated an association between expression of gut taste receptors and weight loss, glycaemia and circulating gut hormones after bariatric surgery are summarised in Table 5.

**Table 5:** Association between expression of taste receptors and weight loss, fat mass, and circulating glucose, lipids and gut hormones after bariatric surgery

**Author** **(year)**	**Surgery**	**Δ Weight**	**Δ Fat mass**	**Glycaemic control**	**Lipid profile**	**Plasma gut hormones**	**Taste receptor analysed**	**Analysis of association between clinical outcome and taste receptor changes**
Pepino et al. (2014) [48]	RYGB LAGB	%TWL RYGB: 20.3 ± 3.0 LAGB: 18.4 ± 2.0	N/A	N/A	N/A	N/A	α-gustducin PLCβ2 T1R3 T1R2 T1R1	No
Nguyen et al. (2014) [49]	RYGB	N/A	N/A	↓ Fasting BGL compared to unoperated controls with obesity ↓ fasting plasma insulin ↑ rise in post-prandial insulin compared to lean controls, but still ↓ compared to obesity controls	N/A	N/A	T1R2 SGLT1	Peak BGL correlated with baseline fasting expression of SGLT1 post-RYGB No significant relationships between baseline or post-infusion T1R2 expression and plasma BGL or 3-OMG concentrations
Steensels et al. (2017) [58]	RYGB	↑ TWL in WT mice compared to α-gustducin-KO mice	↓ total fat pad mass in both genotypes	↑ glucose tolerance in WT but not in α-gustducin-KO mice ↑ rate of rise of BGL following OGTT Pre-RYGB insulin in α-gustducin-KO mice ↓ vs WT mice. ↓ fasting and OGTT-stimulated insulin in WT mice but not α-gustducin-KO mice	N/A	↑ post-prandial PYY in α-gustducin-KO mice but not WT mice ↑ post-prandial GLP1 in both genotypes ↑ total ghrelin in α-gustducin-KO mice but not WT mice	α-gustducin T1R3 SGLT1 LPAR5 FFAR4 FFAR2 FFAR3	Association cannot be interpreted due to differences in the effect of sham surgery between α-gustducin KO and WT mice ↑plasma PYY and ghrelin but not GLP1 are α-gustducin-dependent
Le Gléau et al. (2021) [35]	EGA	↓ body weight	↓ fat mass	↓ fasting and post-OGTT BGL, insulin, C-peptide ↓ HOMA-IR ↑ baseline and 60-min OGTT-stimulated glucagon	N/A	↑ fasting PYY ↑ post-prandial GLP-1 ↓ post-prandial GIP	α-gustducin	No
Jiang et al. (2022) [62]	DJB	N/A	N/A	↓ fasting BGL, ↑ insulin, ↓ HOMA-IR in T2DM rats	N/A		SGLT1 T1R2 T1R3	No
Yu et al. (2020) [68]	SA-DJB	N/A	N/A	<--> fasting BGL ↓ fasting insulin ↓ AUC glucose during Intraperitoneal glucose tolerance test	N/A		FFAR2 FFAR3	No
Hankir et al. (2017) [56]	RYGB	↓ body weight from postoperative week 2 (P < 0.01)	N/A	N/A	N/A	N/A	OEA PPAR-α	No
Fruhbeck et al. (2022) [67]	SG	↓ body weight	↓ total white adipose tissue	<--> Fasting BGL, insulin, HOMA-IR ↑ AUC of BGL during OGTT	↓ triglycerides in DIO but not genetic obesity ↓ total cholesterol in genetic obesity but not DIO ↓ free fatty acids in DIO but not genetic obesity	↓ ghrelin <--> GLP1 <--> GIP	CD36	No
Bueter et al. (2011) [55]	RYGB	↓ body weight	N/A	N/A	N/A	↑ GLP-1 ↑ PYY	T1R2 T1R3	No
Hutch et al. (2020) [66]	SG	↓ body weight in PPAR-α-KO, GPR119-KO, CD36-KO and WT mice ↑ TWL in PPAR-α-KO mice compared to WT mice (but ↑ pre-operative weight in PPAR-α-KO mice)	↓ fat mass in WT, CD36-KO, GPR119-KO and PPAR-α-KO mice	↑ peak BGL at 15 min during OGTT in CD36-KO and WT mice, but more rapidly returned to baseline ↓ 30- and 45-min BGL during OGTT in PPAR-α-KO and WT mice, and 15- and 30-min in GPR119-KO mice ↓ post-prandial BGL in WT and GPR119-KO mice	↓fasting and post-prandial plasma cholesterol in WT, CD36-KO and PPAR-α-KO mice <--> fasting or post-prandial plasma triglycerides in CD36-KO mice	↑ post-prandial GLP-1 in WT and GPR119-KO mice	OEA CD36 GPR119 PPAR- α	CD36, GPR119 and PPAR- α are not required for the reduction in body weight or fat mass, or improved glycaemia and lipidaemia after SG GPR119 is not required for changes to plasma GLP-1 after SG. Other genes were not analysed for association with GLP-1
Yan et al. (2013) [60]	DJB	↓ body weight by 12% 3 days after surgery with slow regain following but never reaching pre-surgical baseline	N/A	↓ AUC of BGL during OGTT by 38%	N/A	N/A	SGLT1	No
Jurowich et al. (2013) [61]	DJB	<--> body weight within 3 weeks post-surgery	N/A	↓ fasting and OGTT BGL in T2DM rats <--> fasting c-peptide	N/A		SGLT1	No
Kaufman et al. (2019) [54]	RYGB	↓ body weight from day 10 post-surgery onwards ↓ body weight by 165g by day 30 post-surgery	N/A	↓ BGL during Intraperitoneal glucose tolerance test at 5min but <--> from 30-120min	↓ postprandial triglycerides ↓ post-prandial cholesterol	N/A	CD36	No
Kim et al. (2015) [59]	DJB	<--> body weight	N/A	↓ BGL <--> insulin	N/A		SGLT1	No
Taqi et al. (2010) [53]	RYGB	↑ mean TWL by 10%	N/A	N/A	N/A	↑ fasting and post-prandial GLP-2 and PYY	SGLT1	No
Ren et al. (2022) [65]	SG	↑ TWL	N/A	↓ AUC BGL in OGTT	N/A	N/A	SGLT1	No
Stearns et al. (2009) [51]	RYGB	↑ TWL immediately post-surgery on liquid diet ↓ weight gain when placed on high fat diet post-surgery	N/A	N/A	N/A	N/A	SGLT1	No
Pérez-Arana et al. (2022) [52]	RYGB	<--> body weight from day 0–32 post-surgery ↓ weight gain from day 32 post-surgery	N/A	<--> AUC BGL and ↑ insulin during OGTT at 3 & 11 weeks post-surgery ↑ AUC BGL and <--> insulin during OGTT at 23 weeks compared to non-operated controls but <--> compared to sham-operated rats	N/A		SGLT1	No
Xia et al. (2019) [63]	SG	↑ TWL by 17.65% in 30 days ↑ weight regain from day 40 to day 60, surpassing sham group by 15.8%	N/A	↓ fasting BGL at 14 days ↓ fasting and AUC BGL at 1 month post-surgery ↑ fasting and AUC BGL at 2 months post-surgery	N/A	N/A	SGLT1	No
Du et al. (2018) [64]	SG	↓ body weight throughout study duration	N/A	↓ fasting and AUC BGL ↓ fasting insulin and HOMA-IR	N/A		SGLT1	No
Jurowich et al. (2015) [69]	IIP	<--> body weight 4 weeks post-surgery ↓ body weight gain following high fat diet post-surgery	N/A	<--> fasting BGL ↓ BGL post-prandially and during OGTT	N/A	↑ post-prandial GLP-1 by 9-fold <--> fasting GLP-1	SGLT1	No
Cavin et al. (2016) [50]	RYGB SG	↑ TWL following both surgery types	N/A	↓ AUC BGL during OGTT	N/A	<--> fasting GLP-1 ↑ post-prandial GLP-1	SGLT1	No

*(%)TWL* (percentage) total weight loss, *RYGB* Roux-en-Y gastric bypass, *LAGB* laparoscopic adjustable gastric banding, *EGA* entero-gastric anastomosis, *DJB* duodenal-jejunal bypass, *SG* sleeve gastrectomy, *SA-DJB* single-anastomosis duodenal-jejunal bypass, *IIP* ileal interposition, *BGL* blood glucose level, *OGTT* oral glucose tolerance test, *AUC* area under the curve, *HOMA-IR* homeostatic model assessment for insulin resistance, *WT* wild type, *KO* knockout, ↓ decrease, ↑ increase, <-> no change, *GLP-1* glucagon-like peptide 1, *GIP* Gastric inhibitory polypeptide, *PYY* peptide YY

Basal alimentary limb SGLT1 mRNA expression positively correlated with peak post-prandial serum levels of glucose, as well as the non-metabolised glucose analogue 3-O-methyl-D-glucose (3-OMG), in humans after RYGB [49]. The same study found no correlation between plasma concentration of these sugars and T1R2 mRNA expression.

SG reduced weight and improved glucose tolerance in mice with whole body knockout of downstream (PPARα) and upstream (GPR119, CD36) signalling targets of OEA [66]. Circulating GLP-1 levels after a mixed meal test increased similarly after SG in both WT and GPR119KO mice compared with respective sham control animals (58). Due to differences in the effect of sham surgery between α-gustducin gene knockout and wild-type mice, the effects of gustducin-mediated taste receptor signalling on body weight, glucose homeostasis and gut hormone secretion after RYGB are unclear [58].

No studies examined relationship between taste receptor expression and lipid profile after bariatric surgery.

## Discussion

**Fig. 3:**
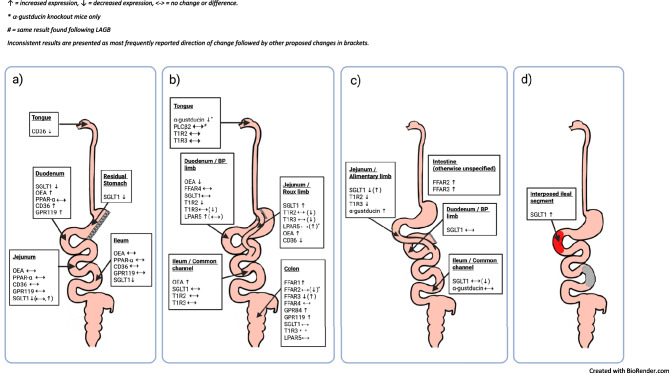
Overview of the changes to expression and translation of taste receptors throughout the gastrointestinal tract after sleeve gastrectomy (**a**), Roux-en-Y gastric bypass (**b**), duodenojejunal bypass or enterogastric anastomosis (**c**) and ileal interposition (**d**)

This is the first systematic review of the effect of bariatric surgery on gastrointestinal taste receptor expression. Overall, the data indicates that changes in mRNA or protein expression of the intracellular taste signalling molecule α-gustducin; sweet taste receptors SGLT1, T1R2, and T1R3; amino acid receptor LPAR5 (GPR92/93); and fatty acid receptors CD36, OEA, FFAR1-3, GPR119, GPR84, occur after all types of bariatric surgery (Fig. 3). Changes to α-gustducin and the sweet and amino acid taste receptors are more commonly reported in intestinal segments that have been surgically repositioned more proximally, such as the alimentary limb after gastric bypass or the interposed ileal segment after ileal interposition surgery. Conversely, changes to fatty acid receptors were more often found in the colon than in the small intestine. Limited data indicate that levels of other taste receptors, including FFAR4, amino acid receptor monomer T1R1; and intracellular taste signalling molecules PLCβ2 and PPAR-α are unaffected by bariatric surgery.

Few studies have examined relationships between taste receptor expression and clinical outcomes of bariatric surgery. Those examining taste preferences have focused on fat. The reduced fat preference observed in mice subjected to SG and RYGB appears to be dependent on CD36 [66] receptors, intracellular intestinal OEA production [56] and PPAR-α activation [56, 66], as disruption of any of these signalling processes by pharmacological blockade or gene knockout negated the effects of bariatric surgery on preference for fat. Conversely, fat receptor GPR119 is not necessary for reduced fat preference after SG [66]. An association between taste receptors and preference for fat has also been reported in non-operated mice, where double-knockout of fat receptors GPR40/FFAR1 and GPR120/FFAR4 prevented the development of fat preference observed in their wild-type or single gene knockout counterparts [20]. Interestingly, this study found no role for CD36 in determining fat preference [20]. A similar gut-brain feeding circuit involving the stimulation of SGLT1 by sugars [20, 24] on CCK-enteroendocrine cells [21] in mice has been reported to be essential in establishing and maintaining the innate mammalian preference sugar over non-nutritive sweeteners. Given that gastrointestinal expression of SGLT1 and CD36, FFAR1-3, GPR119, and GPR84 fatty acid taste receptors change following bariatric surgery, investigation into whether these changes contribute to reduced preferences for sweet and fatty foods after surgery is of interest.

The association between taste receptor expression and post-surgical weight loss is unclear. It is as yet uncertain if changes in taste receptor expression facilitate, result from, or occur independently of changes in food intake, preferences and weight loss. Both oral and post-oral gastrointestinal sweet, fatty acid, umami and bitter taste receptor expression have been shown to correlate with BMI in humans [38, 39, 80, 81], but not all studies agree [34]. No prior study has reported the effect of diet-induced weight loss on taste receptor expression but changes in nutrient intake can acutely alter taste receptor expression. Observational studies in healthy humans have demonstrated that the presence of glucose in the intestinal lumen can acutely alter duodenal expression of sweet taste receptor T1R2 [81]. Short and longer-term consumption of a high-fat diet was associated with reduced expression of oral fatty acid [82] receptors in rodents, whereas humans who followed an 8-week low-fat diet exhibited an increase in oral FFAR4 [83]. After bariatric surgery, most taste receptor expression changes occur in bowel segments newly exposed to incompletely digested nutrients (*see *Fig. 3) [35, 53, 61, 66], suggesting that changes to taste receptor expression after bariatric surgery are likely to result from altered nutrient exposure. Mechanistic preclinical studies that separate changes in diet quality from changes in body weight would further understanding of the potential relationship between gastrointestinal taste receptors and weight loss. Additional studies assessing food preference changes following manipulation of gut taste receptors would provide insight into the influence of the gut mucosa on eating behaviours.

Association between taste receptor expression and glycaemia following bariatric surgery appears to be taste monomer dependent. Fasting SGLT1 expression was reciprocally associated with glycaemia in humans after RYGB [49], in keeping with the known role of SGLT1 in sugar absorption. The lack of association between basal sweet receptor T1R2 expression and glycaemia after bariatric surgery [49] is in line with prior studies conducted in non-operated individuals with and without diabetes [34].

There is substantial heterogeneity of methods between the 23 included studies, including variations in surgery type, interval between surgery and tissue collection, anatomical location and type of receptor examined, and method of receptor analysis. Hence, the variability in results between studies is not surprising. Different changes to mucosal morphology and expression of sweet taste receptors and glucose transporters observed between RYGB and SG indicates that the intestine adapts differently to the two procedures [50]. Diurnal variation in the expression of oral and post-oral gastrointestinal taste receptors has also been observed in several rodent models [84, 85], yet reporting on the timing of tissue harvest is not standard practice. Furthermore, just as post-operative weight and metabolic benefits of bariatric surgery plateau over time [26, 27], the same pattern may occur for structural adaptations induced by these surgeries [50, 63, 67]. Most of the included studies relied on analysis of mRNA to make conclusions about the effect of bariatric surgery on taste receptors, however this does not capture post-transcriptional changes, such as those reported in three studies that analysed both genes and proteins [51, 55, 64].

Further limitations are that several taste receptor molecules were only investigated by a single study, and many have not been analysed in human tissue. No study has investigated the effect of SG on gastrointestinal taste receptors in humans. By including only studies that utilised tissue analysis, this review may have missed studies examining other changes to taste receptors, including functional changes.

## Conclusion

This review examines the effect of bariatric surgery on the expression of taste receptors in the oral cavity and along the gastrointestinal tract. While expressional differences in bitter, sweet, fatty and amino acid receptors as well as intracellular taste signalling molecules occur following bariatric surgery, the results are inconsistent. Changes to the gene or protein expression of intracellular α-gustducin, sweet and amino acid receptors occur most often in intestinal segments surgically repositioned more proximally whereas changes to fatty acid receptors were reported more frequently in the colon than in the small intestine. There is a lack of human studies and paucity of data investigating associations between expressional changes and clinical outcomes of bariatric surgery. Understanding the mechanisms that underlie changes in eating behaviour seen in patients after bariatric surgery will facilitate better understanding of the physiology of these surgeries. It may also provide the opportunity to replicate this effect via non-surgical treatments for obesity, such as the development of medications targeting preference for highly palatable foods or the design of effective flavour agonists able to satisfy cravings for sweet or fatty foods without the associated intake in energy.

## Data Availability

N/A (no original data).

## Abbreviations

*GPCR and GPR*: *G protein-coupled receptor*
*PLCβ2*: *1-Phosphatidylinositol-4,5-bisphosphate phosphodiesterase beta-2*
*TRPM5*: *Transient receptor potential cation channel subfamily M member 5*
*ATP*: *Adenosine triphosphate*
*FFAR*: *Free fatty acid receptor*
*SGLT1*: *Sodium-glucose transporter 1*
*CD36*: *Cluster of differentiation 36, also known as fatty acid translocase*
*GLP-1*: *Glucagon-like peptide 1*
*CCK*: *Cholecystokinin*
*PYY*: *Peptide YY*
*GIP*: *Glucose-dependent insulinotropic polypeptide, also known as Gastric Inhibitory Peptide*
*GLUT2*: *Glucose transporter 2*
*MRI*: *Magnetic resonance imaging*
*PRISMA*: *Preferred Reporting Items for Systematic Reviews and Meta-analyses*
*NIH*: *National Institute of Health*
*RYGB*: *Roux-en-Y gastric bypass*
*LAGB*: *Laparoscopic adjustable gastric banding*
*qPCR*: *Quantitative polymerase chain reaction*
*DJB*: *Duodenal-Jejunal bypass*
*SG*: *Sleeve gastrectomy*
*EGA*: *Entero-gastric anastomosis*
*SA-DJB*: *Single-anastomosis duodenal-Jejunal bypass*
*IIP*: *Ileal interposition*
*mRNA*: *Messenger ribonucleic acid or messenger RNA*
*LPAR5*: *Lysophosphatidic acid receptor 5*
*PPAR- α*: *Peroxisome proliferator-activated receptor - alpha*
*OEA*: *Oleoylethanolamide*
*3-OMG*: *3-O-methyl-D-glucose*
